# Phosgene Gas

**Published:** 1899-07-01

**Authors:** 


### PHOSGENE GAS.

Both surgeons and anæsthetists have long recognised the occasional occurrence of unpleasant symptoms, amounting in some cases to actual poisoning, from the use of chloroform in ill-ventilated rooms, and many samples of chloroform have been, no doubt unjustly, blamed as impure in consequence of such accidents arising. During the past few years, however, it has been shown in a considerable number of instances that the mischief is due to the decomposition of the chloroform vapour which takes place in the presence of a flame—as, for example, a gas flame—and that the injurious material formed by this decomposition is carbonyl chloride, a body discovered by Sir Humphry Davy and by him called phosgene gas. This is now so well recognised that it should be always borne in mind, for it is another and a very strong reason for avoiding the open method of administration when giving chloroform at night in the presence of any artificial illumination except the incandescent electric light. It is, perhaps, possible that even with a Junker inhaler, if the ventilation of the room were very deficient, some small amount of vapour might reach the flame, and thus give rise to the trouble referred to, but if a Junker is properly used, that is if the vapour is delivered during inspiration only, the amount that escapes must be very slight indeed. At any rate some such apparatus, in which vapour is evolved only in proportion as it is required, must, in addition to its other advantages, be the best protection against the occurrence of a form of poisoning which, although it perhaps only occasionally leads to serious results, is no doubt very commonly the cause of the headache and depression which are so often felt both by surgeons and nurses after a long winter's afternoon of theatre work.

